# Genome-wide DNA methylation analysis of CBCVd-infected hop plants (*Humulus lupulus* var. “Celeia”) provides novel insights into viroid pathogenesis

**DOI:** 10.1128/spectrum.00394-24

**Published:** 2025-04-16

**Authors:** Andrej Sečnik, Helena Volk, Urban Kunej, Sebastjan Radišek, Nataša Štajner, Jernej Jakše

**Affiliations:** 1 Department of Agronomy, Biotechnical Faculty, University of Ljubljana, Ljubljana, Slovenia; 2 Plant Protection Department, Slovenian Institute of Hop Research and Brewing 206918, Žalec, Slovenia; University of Florida College of Dentistry, Gainesville, Florida, USA

**Keywords:** *Cocadviroid rimocitri*, CBCVd, hop plant (*Humulus lupulus*), DNA methylation, pathogenesis, viroid

## Abstract

Viroids are small, naked, infectious single-stranded RNA molecules that exploit host factors to replicate. Some viroids have been linked to severe diseases in agricultural crops, including the recent outbreak of *Cocadviroid rimocitri*, previously known as Citrus bark cracking viroid (CBCVd), in hop plants (*Humulus lupulus*). Numerous studies have demonstrated the involvement of viroid-derived RNA in viroid pathogenesis through interactions with RNAi host factors, leading to alterations in gene expression, metabolism, and phenotype. Recent research efforts have also focused on elucidating viroid-induced changes in DNA methylation patterns via the RNA-directed DNA methylation pathway. In this study, we conducted an epigenome analysis of CBCVd-infected hop plants to provide novel evidence supporting the putative role of DNA methylation in CBCVd viroid pathogenesis. Our findings revealed that several genes involved in pathogen interaction pathways, such as MAPK signaling and LRR, exhibit hypomethylation, suggesting that their increased transcription enhances the host’s ability to counteract the pathogen. Intriguingly, genes associated with RNA transcription and encoding key proteins, such as POL II, POL IV, and POL V, display hypermethylation, highlighting the significance of DNA methylation as a defense mechanism.

**IMPORTANCE:**

Viroids are emerging as a substantial threat to various crops; however, our understanding of the molecular mechanisms governing their pathogenesis and the host’s defense remains incomplete. This knowledge gap leaves crop disease management reliant on unsustainable strategies. Our research seeks to address this issue by examining the complex world of infected hop plants. Specifically, we are investigating the DNA methylation processes, providing insights into the less-explored aspects of the host’s response to viroid interaction. Our aim was to unravel the complexities of how viroids influence the molecular landscape within plants and the corresponding host defenses. By understanding these interactions, we hope to provide insights that lead to more sustainable ways to protect crops and keep agriculture resilient against viroid-related threats.

## INTRODUCTION

Viroids are tiny single-stranded RNA molecules that can induce disease in plants. Unlike viruses, they lack a protein coat and consist of only a few hundred nucleotides. Their replication process involves hijacking host plant enzymes and moving into adjacent cells for host colonization (1). The impact of viroids on various crops is of great concern, as they can severely affect the agricultural and socio-economic aspects of specific regions. One such crop is the hop plant (*Humulus lupulus*), which is traditionally cultivated in specific growing regions and primarily used in the brewing industry (2). Recently, *Cocadviroid rimocitri*, previously known as Citrus bark cracking viroid (CBCVd), has emerged as a significant threat to hop production, with its presence detected in Slovenia, Germany, and, most recently, Brazil (3, 4).

Understanding the molecular mechanisms underlying the coordinated response of plants to stress remains an ongoing challenge (5). This response is highly dependent on the nature and intensity of the stress, as well as the specific plant species and its genetic composition. At the molecular level, the stress response involves intricate alterations in the plant’s transcriptional activity. In the context of plant-pathogen interactions, adjustments in DNA methylation patterns and levels can serve as triggers or inhibitors of specific regulatory pathways, consequently leading to diverse morphological, physiological, and biochemical responses (6–9). Epigenetic modifications at the nucleosomal level, such as DNA methylation, post-translational histone modifications, and small RNA interference, play a crucial role in regulating gene expression in a coordinated manner (10).

Viroid-induced stress can have harmful effects on plants, leading to the manifestation of severe disease symptoms (11). Although plants possess innate mechanisms to mitigate stress, the agricultural sector still faces significant challenges due to these effects, resulting in reduced productivity and crop yields. Understanding the physiological impact of these RNA pathogens on their host plants is crucial for developing more efficient and sustainable strategies to combat them. Currently, the available options for controlling severe viroid strains are limited to rigorous crop phytosanitary measures and eradication, as no host genetic resistance or effective chemical treatments have been discovered (12–14). Numerous studies have demonstrated an association between viroid infections and alterations in the plant transcriptome (15–21). Various studies have consistently demonstrated the impact of viroid infection on the regulation of R genes involved in plant defense mechanisms. These include resistance proteins, such as the resistance protein to Tobacco mosaic virus (TMV) (21) and disease resistance protein 1 (RPM1) (16). Additionally, infection influences the expression of resistance gene analogs (RGAs) and other proteins that interact with NBS-LRRs (22–29). Furthermore, leucine-rich repeat (LRR) proteins are associated with jasmonates and ethylene-dependent systemic resistance, further emphasizing their role in plant defense responses (30). In addition, studies have reported the induction of protein kinases, specifically mitogen-activated protein kinase (MAPK) genes, and calcium signaling in relation to viroid pathogenesis (16, 19, 23, 28).

Emerging evidence also suggests that viroids can affect host DNA methylation patterns (31–37) through their interactions with the host RNA-directed DNA methylation pathway (RdDM), facilitated by viroid-derived small interfering RNAs (vd-siRNAs). These findings highlight the potential interference between viroids and host DNA methylation machinery.

A comprehensive understanding of any disease necessitates a systemic biological approach. Epigenetic investigations are rapidly expanding across plant, animal, and human research fields, with a primary focus on unraveling the role of epigenetic changes in biotic stress (38). Studies on viral (39) and bacterial (40) infections in plants have provided insights into the regulatory role of the host methylome as a common immune mechanism in plants (41). High-throughput sequencing has emerged as a favored approach in viroid and virus research due to its ability to generate vast amounts of data, offering deeper insights than conventional methods. However, there remains a scarcity of studies investigating genome-wide DNA methylation changes induced by viroids in host plants.

The objective of this study was to explore genome-wide DNA methylation alterations in hop plants following infection with CBCVd and establish correlations between these epigenetic changes and previously documented transcriptome modifications observed in CBCVd-infected hop plants (20). By doing so, we aim to reveal how plants reprogram their gene activity in response to viroid stress, providing new insights into the regulatory processes involved.

## MATERIALS AND METHODS

### Plant growth

Virus- and viroid-free hop plants of the clonally propagated cv “Celeia” were obtained from the hop nursery at the Slovenian Institute of Hop Research and Brewing, Žalec, Slovenia. To establish viroid infection, 10 plants were inoculated using the Helios GeneGun (Bio-Rad Laboratories, Inc., Hercules, CA, USA) with 360 ng of the dimeric CBCVd construct (GenBank KM211546), following the methods described by (20, 42). To maintain high humidity and prevent desiccation of wounded tissues, plants were enclosed in polyethylene bags and kept in a growth chamber at 25°C with a photoperiod, 16:8 h, light:dark. After 1 week, the plants were transplanted into 4 L pots and transferred to an isolated field plot, enclosed within an insect-proof netting with a mesh size of 1.6 × 1.6 mm. Viroid-free plants were subjected to the same experimental conditions. Fully developed leaves from both viroid-free and infected plants were collected at the phenological stage BBCH 38 from identical sampling sites (4–5 nodes below the apical bud). The viroid-infected plants reached the infection stage of 50 months post-inoculation (mpi). The collected leaves were promptly frozen in liquid nitrogen, powdered in the laboratory, and stored at −80°C. For subsequent analyses, a minimum of three biological replicates were utilized for each experimental group.

### DNA extraction

DNA was isolated from powdered hop leaves using the cetyltrimethylammonium bromide (CTAB) method described by (43). Briefly, 100 mg of powdered hop leaves were homogenized with a pestle in a mortar containing 800 µL of a 2% (wt/vol) CTAB extraction buffer (2% CTAB; 100 mM Tris-HCl; 1.4 M NaCl; 20 mM ethylenediaminetetraacetic acid (EDTA); 0.2% (vol/vol) β-mercaptoethanol) heated to 65°C. The homogenate was incubated at 65°C for 1.5 h with occasional shaking by inverting. After centrifugation at 15,800 *× g* for 10 min, the supernatant was transferred to a new 1.5 mL tube. An equal volume of chloroform/isoamyl alcohol (24:1) solvent mixture was added, thoroughly vortexed, and centrifuged again for 10 min. The supernatant was transferred to a new 1.5 mL tube, and 0.1 vol of 3 M sodium acetate (pH 5.2) and 1 vol of ice-cold isopropanol were added. The mixture was mixed well and then incubated at −20°C for 30 min. After centrifugation for 10 min, the supernatant was removed, and the DNA pellet was washed with 70% ethanol. The pellet was then resuspended in 50 µL TE buffer (10 mM Tris, pH = 8; 1 mM EDTA) and stored overnight at 4°C. The following day, 0.1 vol of RNase A (10 mg mL^−1^) solution was added to the DNA sample to remove RNA, and the sample was incubated overnight at 20°C. The DNA isolation steps were repeated, starting with the addition of an equal volume of the chloroform/isoamyl alcohol solvent mixture. The quality of the isolated DNA was assessed using a NanoVue spectrophotometer (GE Healthcare, Chicago, IL, USA), and its integrity was verified by agarose gel electrophoresis.

### Bisulfite sequencing

The DNA samples were sent to Novogene Europe (Cambridge, UK) for whole-genome bisulfite sequencing (WGBS) using the NGS platform Illumina NovaSeq 6000 platform. Once the DNA samples passed quality control (QC) testing, the genomic DNA, spiked with lambda DNA, was fragmented to a size range of 200–400 bp. The fragmented DNA was then subjected to bisulfite treatment to convert unmethylated cytosines to uracil while leaving methylated cytosines unchanged. Adapters specific to methylation sequencing were ligated to the treated DNA fragments, followed by the synthesis of double-stranded DNA. The library preparation was completed after the size selection and PCR amplification steps.

### Transcriptomic data

NGS data from our previous research (20), publicly available in the Sequence Read Archive under BioProject PRJNA528793, were incorporated into this study to establish associations between differentially expressed genes and differentially methylated genomic regions in CBCVd-infected hop plants. The raw reads were imported into CLC Genomics Workbench 22.0.2 (QIAGEN, Digital Insight, Aarhus, Denmark). Subsequently, the Trim Reads tool was applied to perform trimming with the following parameters: “Trim using quality scores = Yes,” “Quality limit = 0.05,” “Trim ambiguous nucleotides = Yes,” “Maximum number of ambiguities = 2,” “Automatic read-through adapter trimming = Yes,” “Remove 5’ terminal nucleotides = No,” “Remove 3’ terminal nucleotides = No,” “Trim to a fixed length = No,” “Maximum length = 150,” “Trim end = Trim from 3’-end,” “Discard short reads = Yes,” “Minimum length = 20,” “Discard long reads = No,” “Save discarded sequences = No,” “Save broken pairs = No,” and “Create report = Yes.” Next, the RNA-Seq Analysis tool was employed to align the trimmed reads to the hop reference genome (44) using default parameters. The total read counts were then utilized in DESeq2 (45) for differential gene expression analysis, performed in RStudio (4.2.0.).

### Identification of DMRs

WGBS data were imported into the CLC Genomics Workbench (22.0.2) using the Illumina High-Throughput Sequencing Import tool as paired reads. Default parameters were used for import. The paired reads were then mapped to the hop reference genome (44) using the Map Bisulfite Reads to Reference tool. The available genome annotations generated by Transdecoder were utilized in subsequent analyses. The parameter “Directionality” was set to “Directional,” whereas other parameters were kept at their default values. To mitigate the PCR amplification bias introduced during DNA library preparation, the mapped reads were deduplicated using the Remove Duplicate Mapped Reads tool with default parameters. The resulting deduplicated mapped reads were employed to identify differentially methylated regions (DMRs). For this purpose, the Call Methylation Levels tool was utilized. In the “Methylation call settings” window, within the “Read filter” segment, the “Read 2 soft clip” parameter was set to 5. This adjustment accounted for the bias observed in the first few bases of Read 2, where end-repaired sonicated fragments exhibited a propensity toward non-methylation due to unmethylated cytosines. Under the “Methylation detection” segment, the “Methylation context group” was set to “Standard: CpG, CHG, and CHH.” The “Confirm methylation context in reads” option was enabled, and the “Minimum strand-specific coverage” was set to 4. Thus, we retrieved the methylation levels in various genomic regions of the hop genome. In the last window, “Statistical tests and threshold settings,” we finalized the settings to identify the differentially methylated DNA regions (DMRs) in CBCVd-infected hop plants. The “Statistic mode” was set to “ANOVA,” with a maximum *P* value threshold of 0.05. In the “Window thresholds” segment, the “Window length” was set to 100, and in the “Sample thresholds” segment, the “Minimum high-confidence site-coverage” and “Minimum high-confidence site-count” parameters were set to 4. The remaining parameters were kept at their default values. FDR-corrected *P* values for the identified DMRs were calculated using the “BH” method in R (version 4.2.2). Finally, coding sequence annotations were extracted using the “Extract annotation” tool, enabling the identification of hypermethylated and hypomethylated protein-coding genes. Furthermore, the Annotate with Nearby Gene Information tool was employed to identify gene sequences located in proximity to the identified DMRs. Specifically, gene sequences located within a range of 2 kbp upstream or downstream of a DMR were selected for subsequent analysis.

### Analysis of hyper- and hypo-methylated protein-coding genes

To deduce the potential functions of the differentially methylated coding sequence (DMG), the sequences were aligned against the NCBI nonredundant (nr) protein database and the Viridiplantae subset of the NCBI nr database using BLASTx, with a significance cut-off E value of 10^−5^. For homology-based functional annotations of the DMGs, including biological processes, molecular functions, and cellular components, the Blast2GO command line tool (version 1.5.0) (46) was employed to query the sequences. To gain an overview of the gene pathway network and comprehend the higher-level functions and utility of the biological system, the bidirectional best-hit (BBH) method was employed to assign the Kyoto Encyclopedia of Genes and Genomes (KEGG) pathways. This assignment was carried out using the online KEGG Automatic Annotation Server (KAAS) (http://www.genome.jp/kegg/kaas/) (47). Enrichment analysis was conducted to evaluate the enrichment of different gene ontology (GO) categories among the DMGs in comparison to all annotated genes. Annotation was performed using BLASTX hits aligned against the Viridiplantae entries in the NCBI nr database. The topGO package in RStudio (version 4.2.0) was utilized to perform a weighted Fisher’s exact test (*P* value ≤ 0.05) and determine the enrichment of functional categories.

### Total RNA extraction and differential gene expression analysis

Gene expression levels were determined using RT-qPCR. Total RNA was extracted from the sampled material using the Monarch Total RNA Miniprep Kit (New England Biolabs Inc., Ipswich, MA, USA) following the manufacturer’s instructions, including the optional DNase I treatment step. The concentration and quality of the isolated RNA were assessed using a NanoVue spectrophotometer (GE Healthcare, Chicago, IL, USA) and an Agilent Bioanalyzer electrophoresis with the RNA 6000 Nano Kit (Agilent Technologies, Inc., Santa Clara, CA, USA) prior to further analysis. For cDNA synthesis, 2 µg of total RNA and a High-Capacity cDNA Reverse Transcription Kit (Thermo Fisher Scientific Inc., Waltham, MA, USA) were used, following the manufacturer’s instructions. qPCR primers for gene expression analysis were designed using Primer3plus (https://www.bioinformatics.nl/cgi-bin/primer3plus/primer3plus.cgi; accessed January 24, 2023).

The qPCR reactions were conducted using the QuantStudio 5 Real-Time PCR System (Thermo Fisher Scientific Inc., Waltham, MA, USA). To measure gene expression, 1 µL of diluted cDNA (1:100) was amplified in a 6 µL reaction volume containing nuclease-free water, 1X Fast SYBR Green Master Mix (Thermo Fisher Scientific Inc., Waltham, MA, USA), and 300 nM primers (Supplementary file, Table S1). The amplification protocol consisted of initial denaturation at 95°C for 20 s, followed by 40 cycles of denaturation at 95°C for 15 s and annealing/extension at 60°C for 30 s. Melting curve analysis was performed, involving steps of denaturation at 95°C for 15 s, followed by a decrease in temperature to 60°C for 1 min at a rate of 1.76°C s^−1^, and a subsequent increase to 95°C for 15 s at a rate of 0.075°C s^−1^. Relative gene expression values were normalized to the *DRH1* reference gene (48) and calculated using the Pfaffl method (49). Statistical significance (*P* value ≤ 0.05) was assessed using the Duncan multiple range test (50) in RStudio (4.2.0.).

## RESULTS

In the present study, 559.6 Gb were generated by WGBS (Table 1), representing approximately 30× coverage of the estimated hop genome size at 3 Gb. Clean reads were then mapped to the hop reference genome (44), resulting in 92.5, 94.9, 94.7, 94.6, 94.2, and 93.4% mapped reads for CB4_2, CB4_5, CB4_7, VF9_5, VF9_7, and VF9_9, respectively (Table 1). Raw reads were submitted to SRA under BioProject ID PRJNA980399.

**TABLE 1:** Whole-genome bisulfite sequencing (WGBS) data quality and read mapping summary^*a*^^,^*^b^*

Experimental group	Plant number	Raw reads	Raw data	Effective	Q_20_	BS conversion rate	Mapped reads
CBCVd infected	1	626,935,116	94 Gb	99.8%	96.2%	99.8%	92.5%
CBCVd infected	2	656,976,024	98.5 Gb	99.7%	96.0%	99.8%	94.9%
CBCVd infected	3	655,328,676	98.3 Gb	99.7%	96.0%	99.8%	94.7%
Viroid-free	1	614,124,068	92.1 Gb	99.6%	96.7%	99.8%	94.6%
Viroid-free	2	605,688,690	90.9 Gb	99.5%	96.4%	99.7%	94.2%
Viroid-free	3	632,050,090	94.8 Gb	99.8%	96.4%	99.8%	93.4%

^*a*^ The number of raw reads, data size, percentage of total reads used (Effective), read quality, BS conversion rate, and percentage of mapped reads to hop reference genome for every tested hop plant are shown.

^*b*^ Effective: (number of clean reads/number of raw reads) × 100%; Q_20_: (No. of bases with Phred > 20) / (Total No. of bases).

To explore the impact of CBCVd infection on DNA methylation levels in hop plants, we assessed the average number of methylated cytosines in each methylation context for all tested samples. Additionally, the difference between CBCVd-infected and uninfected hop plants provided us with valuable insights into the changes in the DNA methylation level (Table 2).

**TABLE 2:** Percentage (%) of methylated cytosines in different methylation contexts^*a*^

Methylation context	CBCVd-infected hop plants	Viroid-free hop plants	Absolute difference (infected—viroid-free)
CpG	89.7%	89.0%	+0.7%
CHG	77.4%	76.9%	+0.5%
CHH	8.3%	8.4%	–0.1%
Any	26.9%	26.7%	+0.2%

^*a*^ C, cytosine; G, guanine; H, adenine, cytosine, or thymine.

Consistent with expectations, the results of the two-tailed *t* test revealed no significant difference in the global genomic DNA methylation level between infected (26.9%) and viroid-free hop plants (26.7%) (Table 2). Likewise, no significant differences were observed in the DNA methylation level of the whole genome, considering methylation contexts (CpG, CHG, and CHH) (Table 2). These findings align with the preliminary results obtained using HPLC-UV analysis (37), indicating that the infection of hop plants with CBCVd has no discernible impact on the overall DNA methylation level of the hop plant genome, regardless of the methylation context.

**Fig 1:**
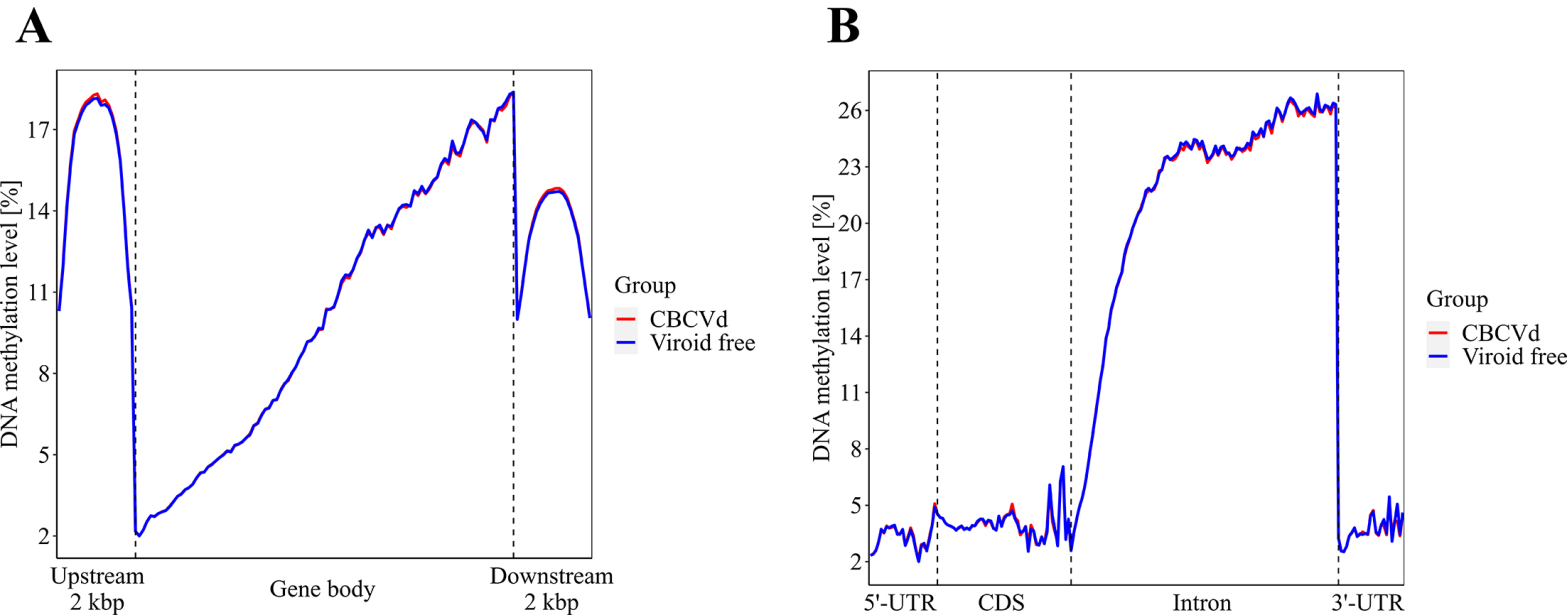
Distribution of DNA methylation levels in various genomic regions in CBCVd-infected (red) and viroid-free (blue). To show the low-resolution DNA methylation level profile, the genome was divided into 100 bp windows. (**A**) Comparison of DNA methylation level distribution of the gene body and flanking up- and downstream regions. (**B**) Comparison of the DNA methylation level distribution of the gene body functional regions, such as 5′-UTRs, CDS, introns, and 3′-UTRs.

To explore the potential regulatory role of gene flanking regions, we extended our analysis to investigate the distribution of DNA methylation levels within gene bodies and in regions spanning up to 2 kbp upstream and downstream of genes (Fig. 1). Consistent with expectations, we observed distinct methylation patterns in the flanking regions compared with the gene bodies, regardless of infection status. Although visually higher DNA methylation levels were observed in these functional regions of CBCVd-infected hop plants (Fig. 1), these differences were minimal and not significant.

**Fig 2:**
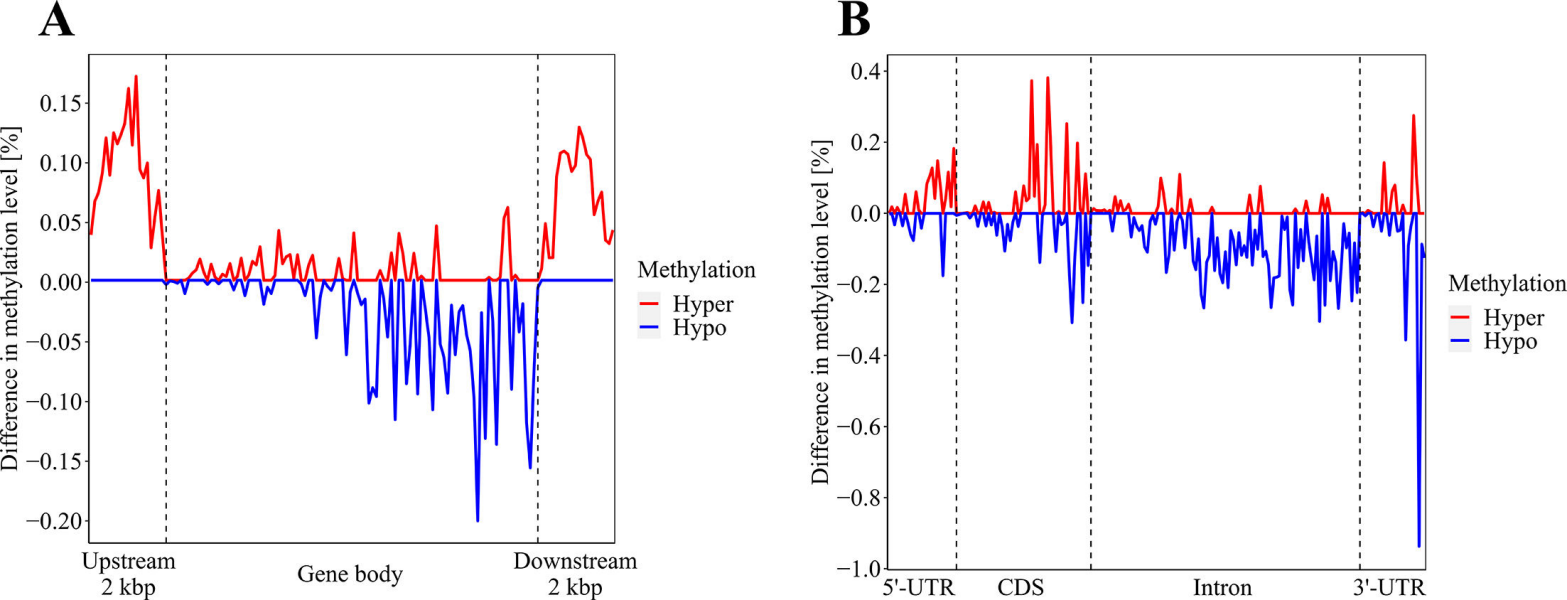
Distribution of DNA methylation level differences in various genomic regions of CBCVd-infected hop plants, represented as hypermethylation (red) and hypomethylation (blue) compared with viroid-free hop plants. The genome was divided into 100 bp windows to visualize the low-resolution DNA methylation profile. (**A**) Comparison of DNA methylation level distribution in the gene body and flanking upstream and downstream regions. (**B**) Comparison of DNA methylation level distribution in gene body functional regions, including 5′-UTRs, CDS, introns, and 3′-UTRs.

Given the relatively minor disparity in DNA methylation levels between CBCVd-infected and viroid-free hop plants, Fig. 2 primarily shows the differential DNA methylation patterns observed in CBCVd-infected hop plants.

In the functional regions depicted in Fig. 1A and 2A, we conducted a two-tailed *t* test to assess the significance of DNA methylation levels based on the methylation context. Our findings revealed a significant increase (*P*- value ≤ 0.05) in DNA methylation levels for the CHH context in both flanking regions of genes in CBCVd-infected hop plants (7.65% vs 7.10% and 5.66% vs 5.29%, respectively). This observation is particularly intriguing as CHH methylation is known to be influenced by Domains Rearranged Methyltransferase 2 (DRM2) through the RNA-directed DNA methylation pathway (51). Conversely, we did not identify any significance in DNA methylation levels within genes or in the flanking regions across other methylation contexts in CBCVd-infected hop plants. Following the same approach as described earlier, we further investigated the variations in DNA methylation level distributions within functional regions of the gene body, namely the 5'-untranslated region (5′-UTR), coding sequence (CDS), introns, and 3'-untranslated regions (3′-UTR), as illustrated in Fig. 1B and 2B. Similar to the gene bodies and the flanking regions, the DNA methylation levels profile in these functional genomic regions were quite similar for the CBCVd-infected and viroid-free hop plants. Notably, the intron regions exhibited the highest levels of DNA methylation in both CBCVd-infected and viroid-free hop plants. This was followed by the coding sequences, the 3′-UTR, and the 5′-UTR. Moreover, when considering the overall DNA methylation levels within the functional regions of the gene body, there were no significant differences observed between CBCVd-infected and viroid-free hop plants, regardless of the methylation context. However, upon closer examination of specific methylation contexts, we identified significantly different results. Specifically, the DNA methylation levels in the CpG and CHG contexts within the CDS, as well as in the CHG and CHH contexts within the 3′-UTR, were higher (*P* value ≤ 0.05) in CBCVd-infected hop plants compared with viroid-free plants (24.72% vs 23.56% for CpG in CDS, and 2.20% vs 2.06% for CpG in 3′-UTR). Additionally, in the CHG context, the DNA methylation levels in CBCVd-infected hop plants were higher than in viroid-free plants (2.70% vs 2.53% for CHG). Similarly, in the CHH context, the DNA methylation levels were slightly higher in CBCVd-infected hop plants compared with the viroid-free group of plants (0.75% vs 0.72% for CHH). These findings suggest that the influence of CBCVd on DNA methylation levels in the hop genome is partially related to the specific methylation context, affecting part of the functional gene body and functional regions within the gene body.

The whole-genome analysis conducted in this study identified a total of 2,196,528 DMRs with a size of 100 bp, demonstrating statistical significance (*P* value ≤ 0.05) and a size of 100 bp. To refine the data set, we applied *P* value filtering with FDR correction (*P* value ≤ 0.05), resulting in 1,967,901 DMRs (Table 3; Supplementary File Table S2). These DMRs were categorized as hypermethylated or hypomethylated based on a comparison of DNA methylation levels between CBCVd-infected and uninfected hop plants. Given the total length of the hop genome, the DMRs represented 5.3% of the whole genome. Specifically, we identified 853,404 hypermethylated DMRs and 1,114,497 hypomethylated DMRs (Table 3). To further refine our analysis, we focused on DMRs that exhibited an absolute difference in DNA methylation rate of ≥15%, regardless of the methylation context. This filtering step resulted in a final set of 9,661 hypermethylated DMRs and 11,013 hypomethylated DMRs. Among these DMRs, we specifically extracted those that overlapped with protein-coding regions in the hop genome. This selection yielded 176 hypermethylated protein-coding genes and 142 hypomethylated protein-coding genes, which were subsequently employed for gene set enrichment analysis. A detailed list of these genes, including their chromosome location, genomic region, unique identifier, and UniProt annotations, is provided in (Supplementary File, Tables S3 and S4).

**TABLE 3:** Overview of DMRs detected in CBCVd-infected hop plants. C: cytosine; G: guanine; H: adenine, cytosine or thymine

Methylation context	Hyper-DMRs	Hypo-DMRs	Total	Hyper-DMRs (≥15%)	Hypo-DMRs (≥15%)
CpG	244,868	269,603	514,471	1,646	1,871
CHG	250,847	325,604	576,451	5,745	7,748
CHH	357,689	519,290	876,979	2,270	1,394
Total	853,404	1,114,497	1,967,901	9,661	11,013

**Fig 3:**
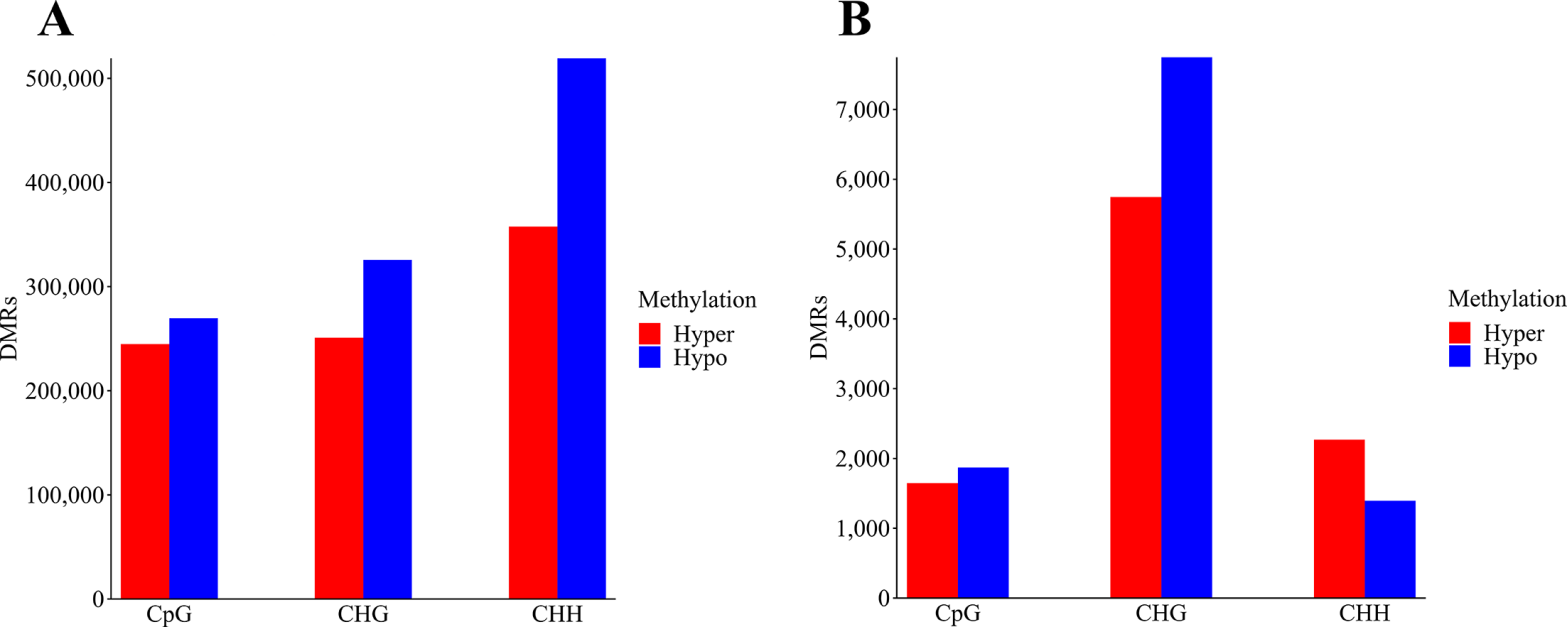
Distribution of DMRs according to methylation context in the genome of CBCVd-infected hop plants. (**A**) Total DMRs. (**B**) DMRs with at least a 15% absolute difference in DNA methylation levels.

Initially, we examined the distribution of DMRs according to the methylation context, as depicted in Fig. 3. The overall count of hypomethylated DMRs surpassed that of hypermethylated DMRs, regardless of the methylation context (Table 3; Fig. 3A). Notably, the majority of DMRs were detected in the CHH methylation context, followed by CHG and CpG (Table 3; Fig. 3A). However, the exclusion of a large number of DMRs exhibiting a DNA methylation level difference of less than 15% slightly affected their distribution (Table 3; Fig. 3B). Nevertheless, the total count of hypomethylated DMRs remained higher than that of hypermethylated DMRs (Table 3; Fig. 3B). Interestingly, among the different methylation contexts, the CHG context displayed the greatest number of DMRs, followed by CHH and CpG. Remarkably, within the CHH context, there was a higher count of hypermethylated DMRs compared with hypomethylated ones (Table 3; Fig. 3B).

**Fig 4:**
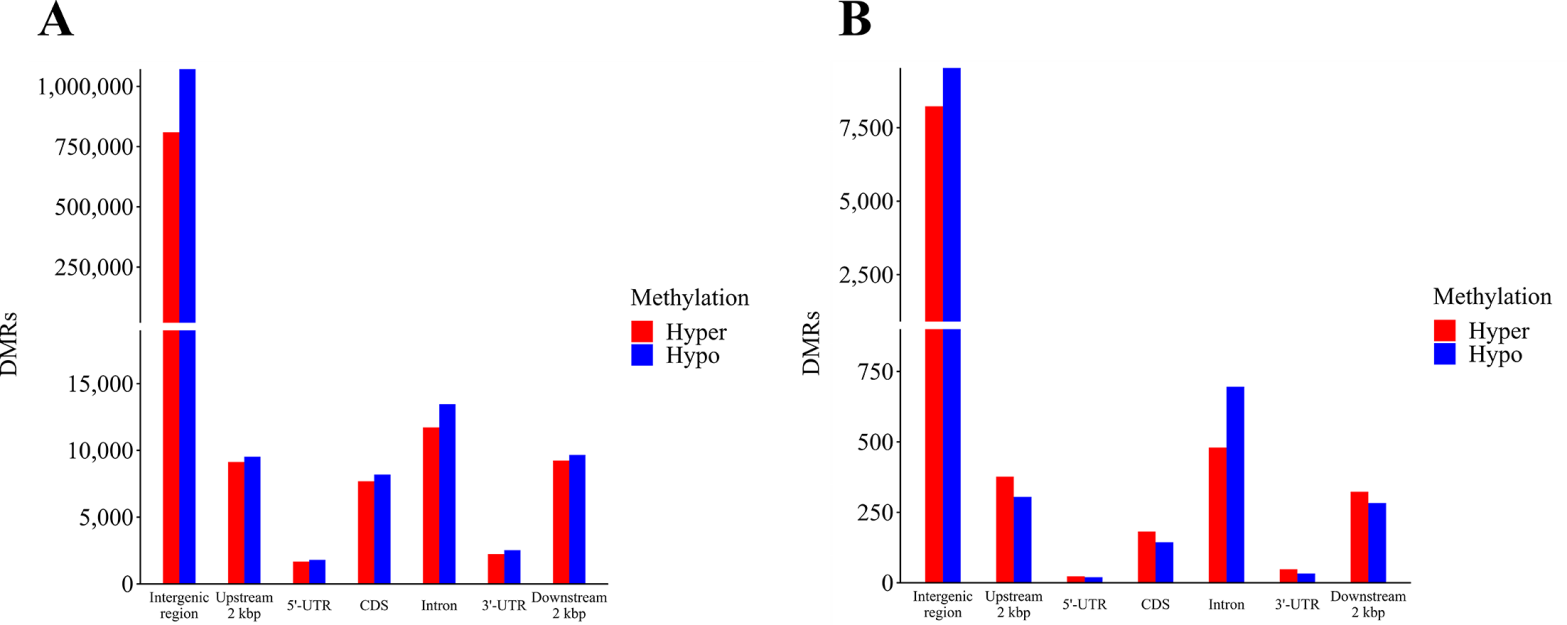
Distribution of DMRs across various genomic regions, including intergenic regions, Upstream 2 kbp, 5′-UTRs, CDS, Introns, 3’UTRs, and Downstream 2 kbp. (**A**) Total DMRs in each genomic region. (**B**) Number of DMRs remaining after applying a filter for the absolute difference in the DNA methylation level of 15%.

We then investigated the distribution of DMR regions across various genomic regions of the hop genome, regardless of the methylation context (Fig. 4). These regions encompassed intergenic regions, as well as the distinct components mentioned above.

A total of 1,863,617 DMRs (94.7%) were found within intergenic regions, whereas 104,284 DMRs (5.3%) were distributed across the functional regions of the genome (Fig. 4A). The cumulative length of DMRs in intergenic regions accounted for 5.4% of the total intergenic segment of the hop genome, whereas DMRs in functional regions represented 4.3% of the length of the functional part of the hop genome. Notably, the count of hypomethylated DMRs surpassed that of hypermethylated DMRs across all genomic regions (Fig. 4A). By imposing a criterion of at least a 15% difference in the DNA methylation level, 98.9% of all DMRs were discarded, resulting in a final set of only 20,674 DMRs. Among these, 17,760 (85.9%) were situated in intergenic regions, whereas 2,914 (14.1%) were located within functional parts of the hop genome, representing an 8.8% decrease or increase compared with the data set prior to the 15% threshold. Furthermore, alterations in the ratio of hypermethylated to hypomethylated DMRs were observed in certain functional genomic regions. This effect was particularly pronounced when considering that the cumulative length of intergenic DMRs (≥ 15%) represented a mere 0.05% of the total intergenic segment, whereas the cumulative length of DMR regions (≥ 15%) within functional regions accounted for 0.12% of the total length of the functional genomic regions. Except for introns (Fig. 4B), hypermethylated DMRs predominated over hypomethylated DMRs in all functional regions of the genome. Finally, 176 hypermethylated and 142 hypomethylated protein-coding genes were identified within the protein-coding segment of the genome (Fig. 4B), which were subsequently utilized for Gene Set Enrichment Analysis.

**Fig 5:**
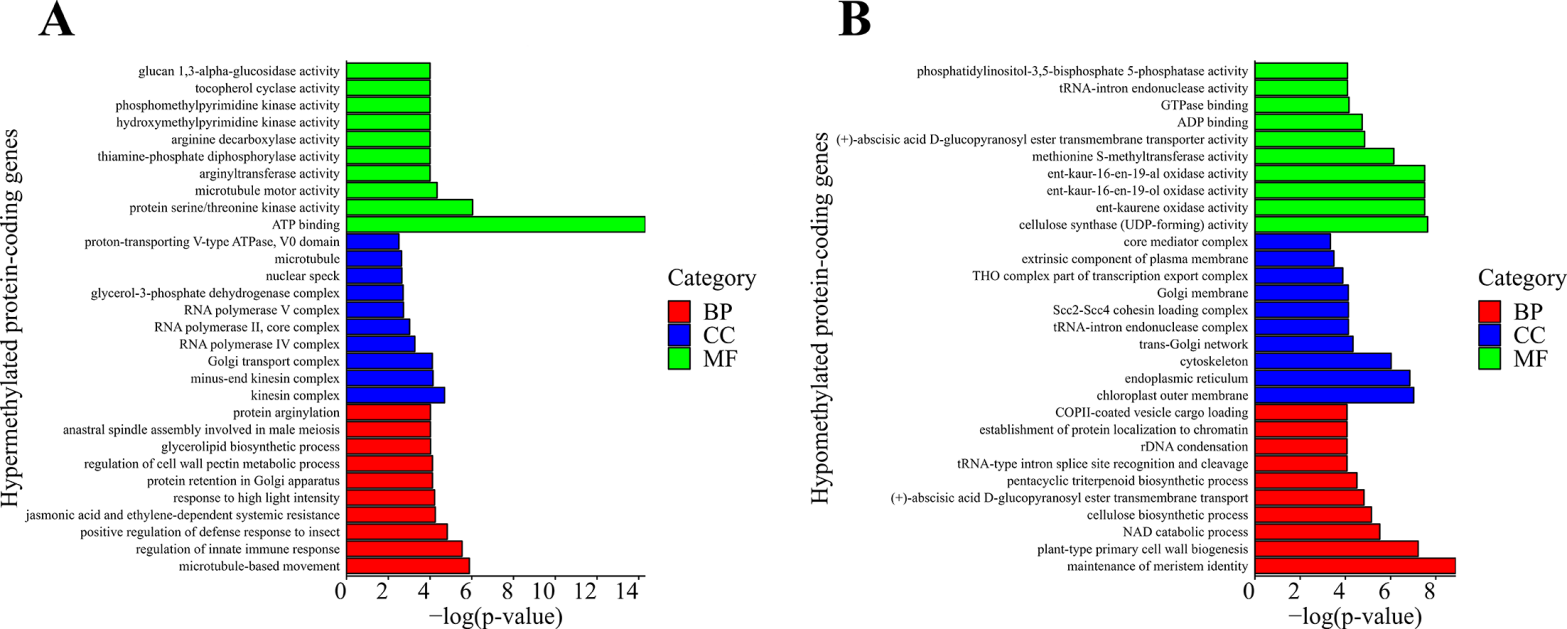
GO analysis of protein-coding genes, showing the top 10 significantly enriched GO terms for (**A**) hypermethylated genes and (**B**) hypomethylated genes. GO terms are categorized into biological processes (BP), cellular components (CC), and molecular functions (MF).

To gain insight into the implications of DNA methylation in CBCVd-infected hop plants, we conducted a gene ontology analysis. The differentially methylated protein-coding genes (DMGs) were categorized into three major groups: biological processes (BP), cellular components (CC), and molecular functions (MF). Our findings revealed a significant enrichment (*P* value ≤ 0.05) of specific GO terms in both hypermethylated and hypomethylated DMGs. Among the hypermethylated DMGs, 92, 20, and 46 GO terms were overrepresented in the BP, CC, and MF categories, respectively. Similarly, the hypomethylated DMGs exhibited enrichment in 81 BP terms, 32 CC terms, and 52 MF terms. Notably, hypermethylated DMGs were associated with processes such as microtubule-based movement (GO:0007018), kinesin complex (GO:0005871), and ATP binding (GO:0005524) (Fig. 5A). Conversely, hypomethylated DMGs were linked to processes such as maintenance of meristem identity (GO:0010074), chloroplast outer membrane (GO:0009707), and cellulose synthase (UDP-forming) activity (GO:0016760) (Fig. 5B).

**Fig 6:**
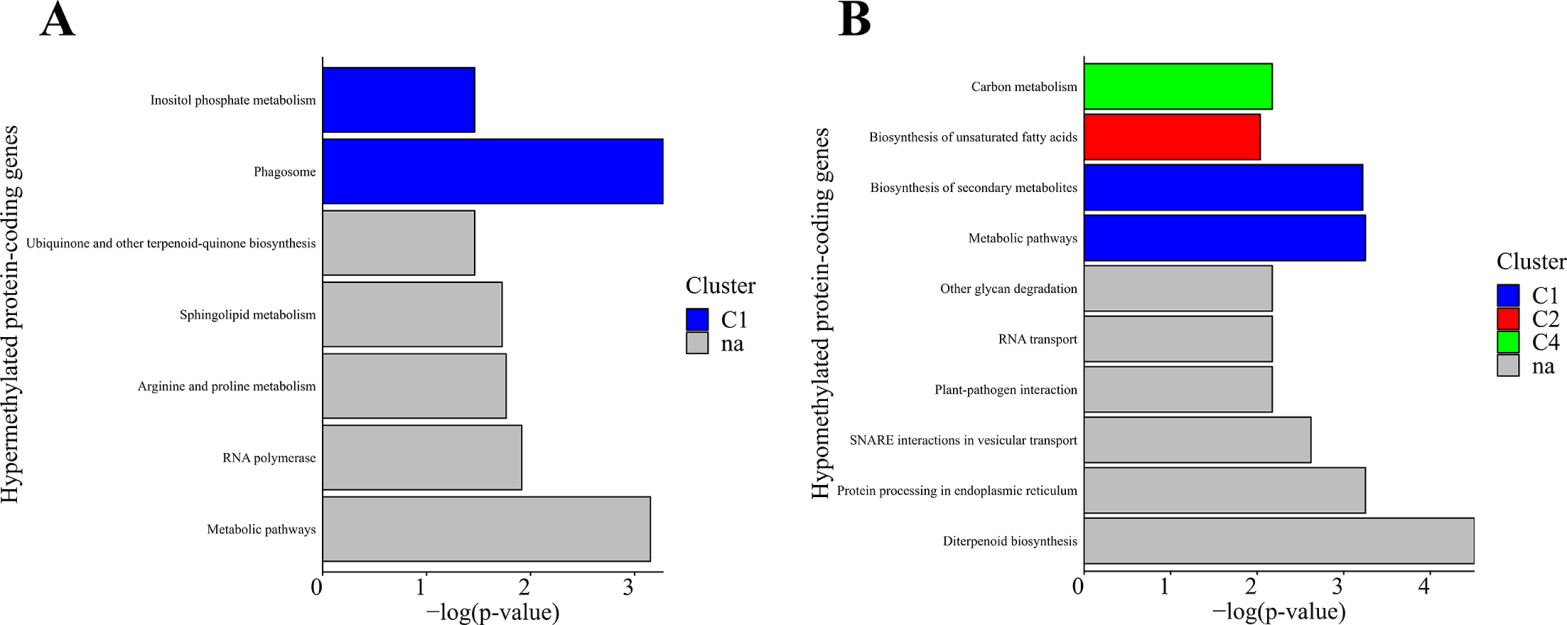
KEGG pathway analysis of protein-coding genes, showing the top 10 significantly enriched GO terms for (**A**) hypermethylated DMGs and (**B**) hypomethylated DMGs.

We extended our analysis by incorporating KEGG analysis to gain insights into the underlying biochemical pathways associated with hyper- and hypo-methylated DMGs. Among the hypermethylated protein-coding genes, we identified seven KEGG pathways that were significantly enriched (corrected *P* value ≤ 0.05) (Fig. 6A). These pathways included phagosome-related processes, metabolic pathways, RNA polymerase activity, and other metabolic activities. Analysis of the hypomethylated DMGs revealed 19 significantly enriched KEGG pathways (corrected *P* value ≤ 0.05). These pathways encompassed diterpenoid biosynthesis, plant-pathogen interactions, nitrogen metabolism, and various amino acid metabolism pathways (Fig. 6B). Additionally, these genes exhibited involvement in RNA transfer and interaction with soluble N-ethylmaleimide-sensitive factor attachment protein receptors (SNAREs) in vesicular transport, DNA replication, and glycan degradation processes.

In line with the transcriptomic analysis by (20) on hop viroid infections, we integrated transcriptomic data into our study to establish potential connections between DMGs and their corresponding upregulated and downregulated genes (DEGs) in response to CBCVd infection in hop plants. Using DESeq2 analysis, we identified 2701 protein-coding genes that exhibited significant differential expression (FDR *P* value ≤ 0.05). For further investigation, we focused on genes with a fold change of at least 2, resulting in a subset of 1212 upregulated DEGs and 1,489 downregulated DEGs.

**Fig 7:**
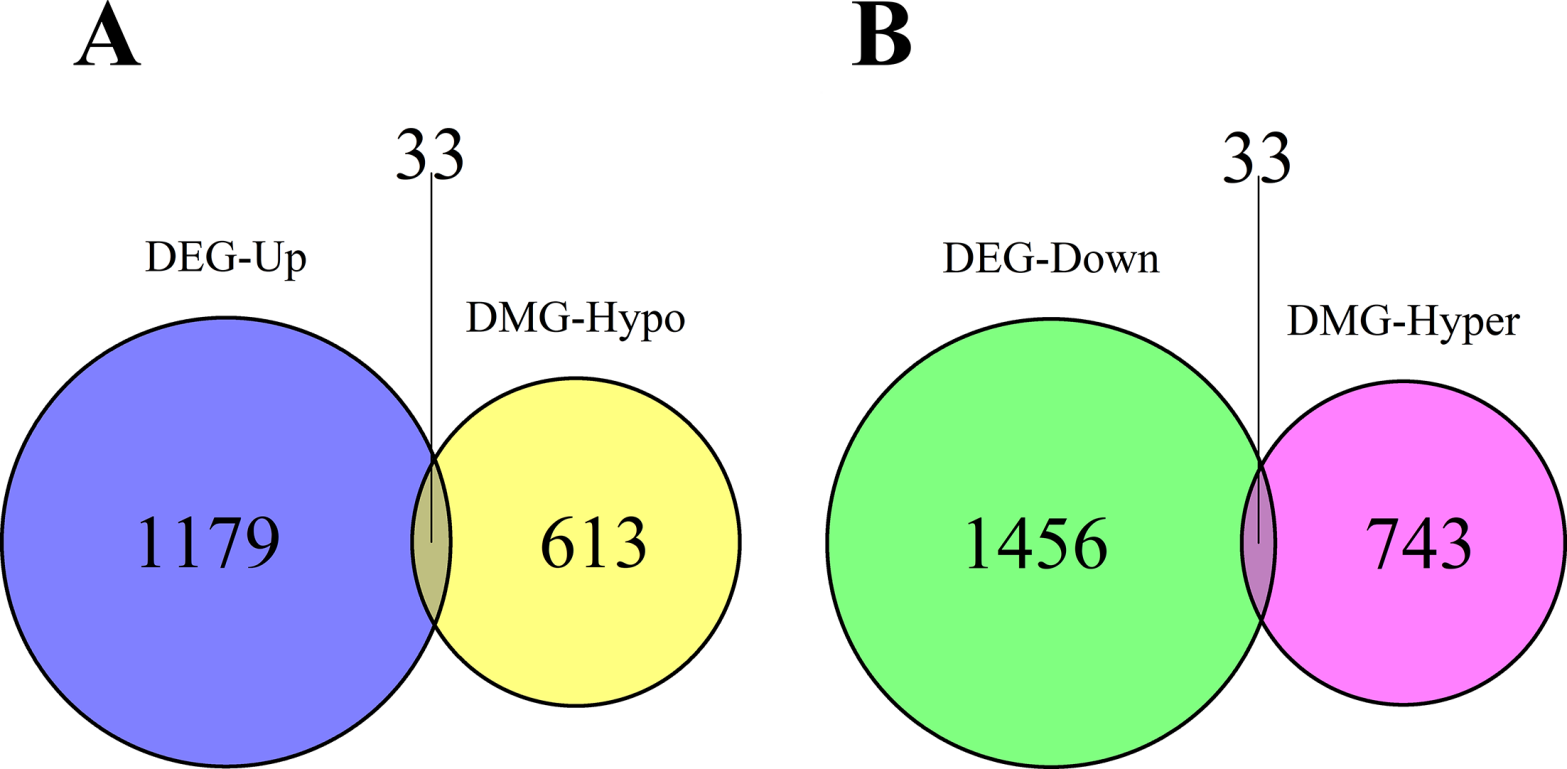
Venn diagram illustrating the relationship between RNA-seq and BS-seq data. (**A**) Overlapping sets of upregulated protein-coding genes (1212) and protein-coding genes associated with hypomethylated DNA (646). (**B**) Overlapping set of downregulated protein-coding genes (1489) and protein-coding genes associated with hypermethylated DNA (776).

Given the putative relationship between DNA methylation and gene expression regulation, we classified genes into two groups based on their DNA methylation status and expression levels (Fig. 7). The first group included hypomethylated DMGs and upregulated DEGs (Fig. 7A), as well as genes that were not necessarily hypomethylated but were surrounded by hypomethylated flanking regions (Fig. 7A). Similarly, the second group consisted of hypermethylated DMGs and downregulated DEG, as well as genes that were not necessarily hypermethylated but were located within hypermethylated DMRs (Fig. 7B). Applying these criteria, we identified 33 candidates in each group (Fig. 7), accounting for only 2.6% of all DEGs (2701). Nevertheless, these criteria allowed us to consider the potential regulatory role of the gene flanking regions.

**Fig 8:**
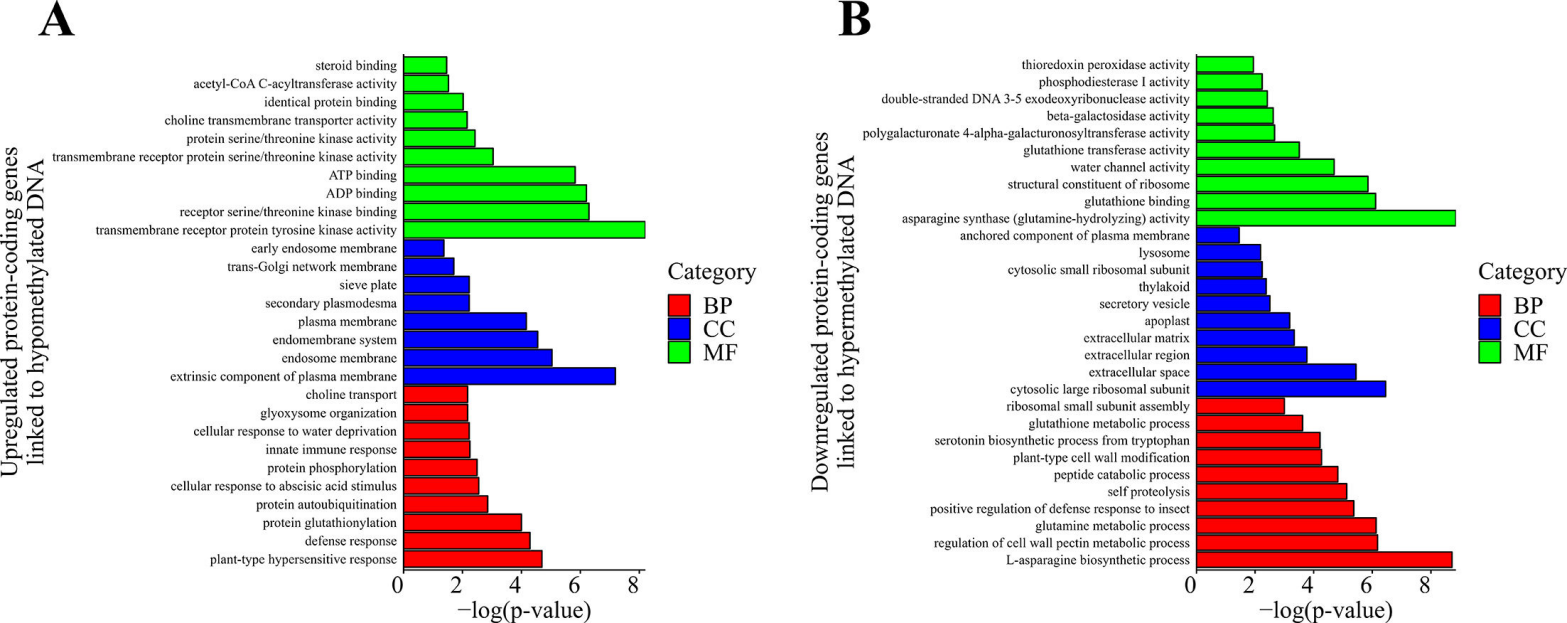
GO analysis of differentially expressed protein-coding genes linked to differential DNA methylation, showing the top 10 significantly enriched GO terms for (**A**) upregulated DEGs linked to hypomethylated DNA and (**B**) downregulated DEGs linked to hypermethylated DNA. GO terms are categorized into biological processes (BP), cellular components (CC), and molecular functions (MF).

The GO analysis of the two gene groups yielded a significant enrichment of GO terms. In the first group, we observed significant enrichment of 25 BP, 8 CC, and 12 MF GO terms (*P* value ≤ 0.05), whereas the second group showed significant enrichment of 21 BP, 11 CC, and 18 MF GO terms (*P* value ≤ 0.05). The enriched processes in the first group included plant hypersensitivity response (GO:0009626), extrinsic component of plasma membrane (GO:0019897), and transmembrane receptor protein tyrosine kinase activity (GO:0004714) (Fig. 8A). The second group exhibited enrichment in processes such as the L-asparagine biosynthetic process (GO:0070981), cytosolic large ribosomal subunit (GO:0022625), and asparagine synthase (glutamine-hydrolyzing) activity (GO:0004066) (Fig. 8B).

**Fig 9:**
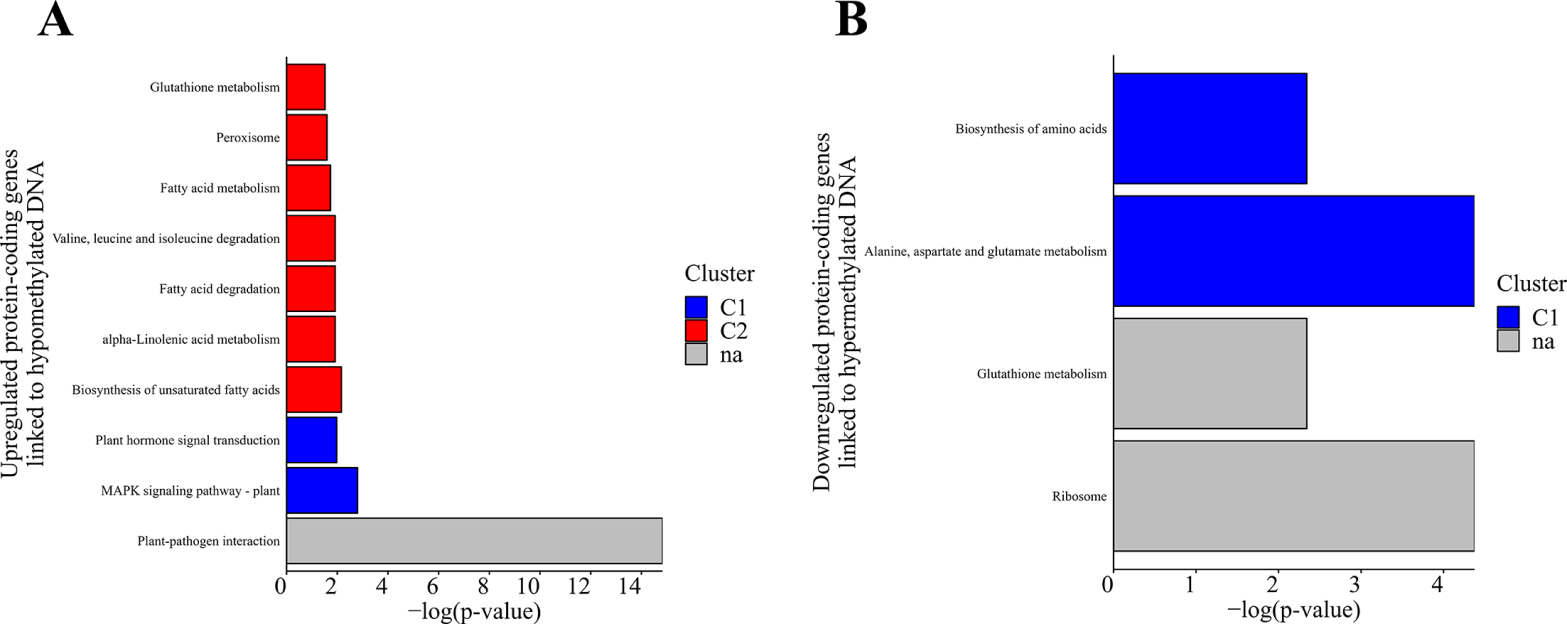
KEGG pathway of differentially expressed protein-coding genes linked to differential DNA methylation, showcasing the top 10 significantly enriched GO terms for (**A**) upregulated DEGs linked to hypomethylated DNA and (**B**) downregulated DEGs linked to hypermethylated DNA.

Based on the protein-coding genes identified in the previous groups, we further conducted a KEGG pathway analysis. The first group, consisting of 33 protein-coding genes, exhibited significant enrichment in 10 biochemical pathways (*P* value ≤ 0.05), whereas the second group, comprising an equal number of protein-coding genes, showed involvement in four significantly enriched pathways (*P* value ≤ 0.05) (Fig. 9). As expected, the protein-coding genes in the first group were primarily associated with plant-pathogen interactions and signaling pathways, including the MAPK signaling pathway and signal transduction via plant hormones (Fig. 9A). Enrichment was also observed in metabolic pathways involving fatty acids and certain amino acids, as well as glutathione metabolism and peroxisome-related processes. In the second group of protein-coding genes, enrichment was identified in biochemical pathways related to amino acid and glutathione metabolism (Fig. 9B). Notably, several pathways associated with ribosomal processes were also enriched.

To validate the gene expressions observed in the RNA-seq data, we performed RT-qPCR analysis on a carefully selected group of 11 genes in the CBCVd-infected hop plants. Gene selection was based on their significant involvement in enriched GO terms or KEGG pathways, indicative of their biological relevance in the context of CBCVd infection. To mitigate any potential selection bias, two additional up- and down-regulated DEGs were randomly included in the RNA-seq data set. Thus, a total of 13 genes were assayed using RT-qPCR (Supplementary File, Table S5).

**Fig 10:**
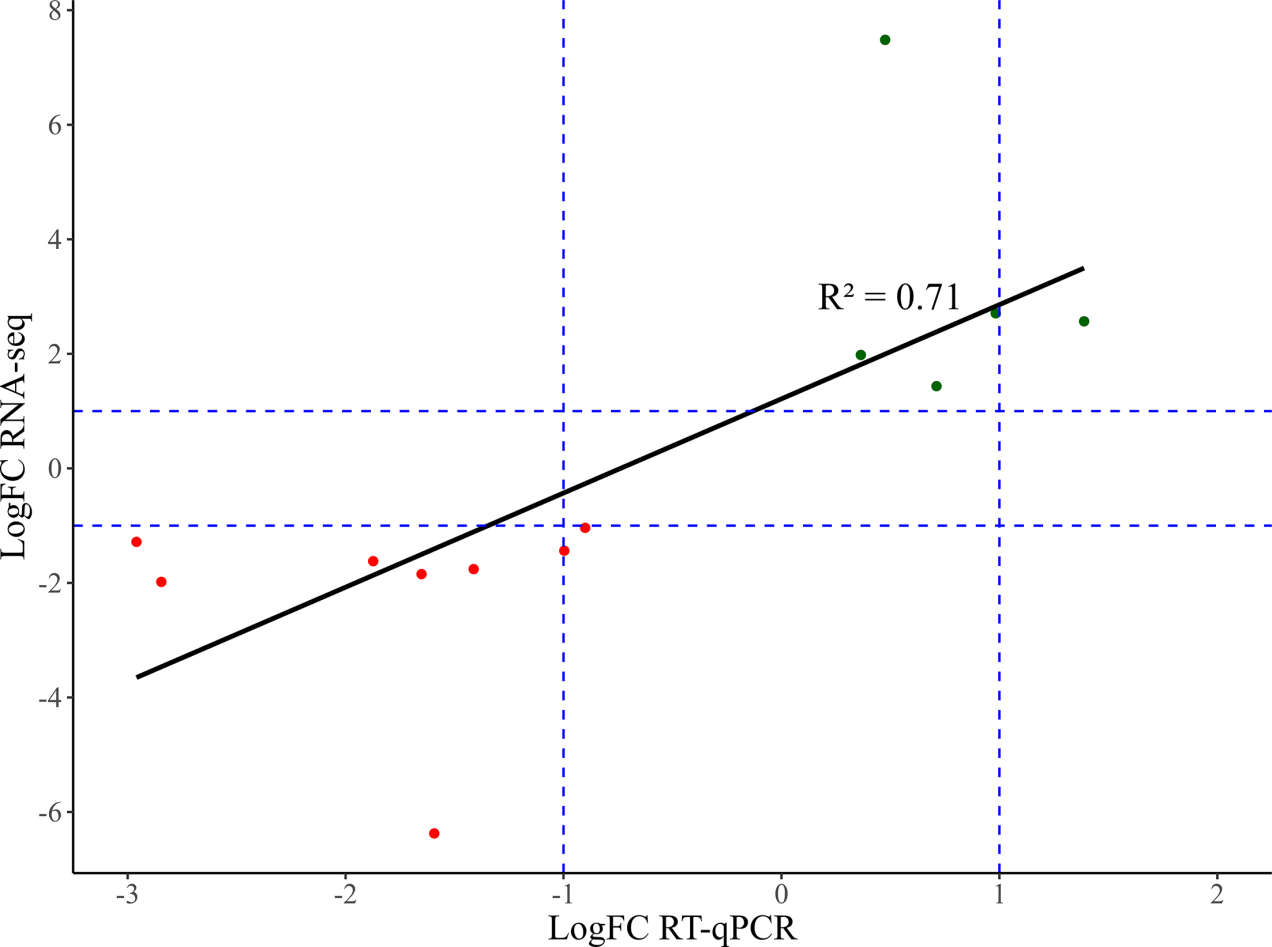
Correlation between RT-qPCR and RNA-seq analysis for DEGs. Positive values (green) and negative values (red).

As depicted in Fig. 10, the Pearson’s correlation coefficient (R2) between the RT-qPCR data and the RNA-seq data was 0.71. RT-qPCR analysis was performed on separate plants, which may introduce inherent biological variability. Detailed information regarding the relative expression of the selected 13 genes is available in Supplementary File 1 (Table S6). Despite this, the correlation coefficient indicates relatively close agreement between the two data sets, affirming the reliability of the RNA-seq data.

## DISCUSSION

Viroids thrive by exploiting the host’s molecular machinery and manipulating its regulatory networks to their advantage. The relationship between DNA methylation and viroids suggests that alterations in host regulatory pathways occur during infection. However, the understanding of how viroids impact host DNA methylation remains limited, with only a few studies investigating the methylation status of specific DNA regions. In our study, we aimed to overcome these limitations by conducting genome-wide DNA methylation profiling of CBCVd-infected hop plants. Furthermore, we incorporated transcriptomic data from infected hop plants obtained from a previous study (20) to gain a comprehensive understanding of the molecular changes associated with CBCVd infection. This study is a continuation of our previous research on cytosine methylation changes in viroid-infected hop plants, as part of a broader effort to comprehend the impact and severity of CBCVd in hop plants. By building upon our earlier work (37), we aimed to deepen our understanding of the molecular mechanisms underlying the effects of CBCVd infection on hop plants, particularly regarding DNA methylation.

Following pathogen infection in plants, transcriptomic changes commonly occur within a few hours preceding epigenetic alterations (52). These transcriptomic changes reflect the plant’s dynamic response to the pathogen and involve the modulation of gene expression. Notably, genes associated with pathogen recognition and defense, including pathogenesis-related (PR) proteins, are upregulated, whereas genes involved in growth and development are often downregulated as part of the plant’s defense response.

The first observations of such changes in response to viroid infection were reported in *Gynura aurantiaca* (53), where the presence of CEVd resulted in significant alterations. In contrast, epigenetic changes exhibit a higher degree of stability, often manifesting gradually over hours or days (54, 55). A recent study has elucidated the temporal dynamics of transcriptomic and epigenetic alterations in cucumber plants following infection with *Hostuviroid impedihumuli*, previously known as Hop stunt viroid (HSVd) (56). This study, like previous research (35, 36, 57), provides an overview of the infection process, capturing the epigenetic and transcriptomic landscape at a specific stage. To gain a more comprehensive understanding of the dynamic changes during infection, future studies should include additional time points to provide deeper and more relevant scientific insights.

Previous research has established that DNA demethylation plays a crucial role in plant immune responses, influencing the transcriptional activation of defense genes (58). In the context of plant-pathogen interactions, the hypomethylation of genes associated with plant defense responses is particularly significant, as it facilitates their transcriptional activation and enhances the plant’s ability to defend itself against pathogens. Conversely, the hypermethylation of genes typically leads to partial or complete silencing of their transcription, impairing the plant’s defense mechanisms (59). However, it is important to note that while differential DNA methylation is an important epigenetic factor in gene regulation in plants, it is not the sole determinant (60).

In plants, cytosine methylation predominantly occurs in the symmetric contexts of CpG and CHG, as well as in the asymmetric context of CHH (where H represents A, C, or T). Similar to other plant species (61), the levels of 5-mC were highest in the CpG context compared with the CHG and CHH contexts (Table 2). Considering the disparity in size between the studied organisms, it may appear improbable for CBCVd to substantially impact DNA methylation at the genomic level. However, our previous research on *Cocadviroid latenshumuli* (HLVd, Hop latent viroid) and the co-infection of CBCVd, HLVd, and HSVd demonstrated contrasting results (37). In CBCVd-infected hop plants, we observed an increase in DNA methylation levels within the gene flanking regions (Fig. 2A), specifically in the asymmetric CHH context. This is interesting because the regulation of methylation in said context is primarily influenced by RdDM (51, 62), suggesting its interference with CBCVd (63). Moreover, we observed hypermethylation in the protein-coding sequences, predominantly in the CpG and CHG contexts, as well as in the 3′-UTRs, primarily in the CHG and CHH contexts (Fig. 2B). These findings indicate that CBCVd infection elicits subtle and finely regulated alterations in the hop genome’s DNA methylation patterns.

In this study, we identified nearly 2 million DMRs (Supplementary File, Table S2) that hold biological significance in CBCVd-infected hop plants. To ensure the robustness of our analysis, we performed a stringent filtering step, excluding approximately 99% of the DMRs that exhibited a difference in DNA methylation level of less than 15% between CBCVd-infected and control hop plants (Supplementary File, Table S2). Consequently, less than 1% of the remaining DMRs were retained, corresponding to 176 hypermethylated and 142 hypomethylated protein-coding genes (Supplementary File, Table S2). These results suggest that CBCVd infection impacts the DNA methylation patterns of specific genes. Our analysis of transcriptomic and epigenomic data identified a relatively small subset of genes—only 66 out of more than 2,000 DEGs—that exhibited concurrent differential DNA methylation. This observation suggests that other epigenetic factors and additional regulatory mechanisms contribute to the modulation of gene expression. Changes in DNA structure and the activity of long non-coding RNAs (lncRNAs) also exert influence over differential gene expression. Moreover, DNA methylation plays a crucial role in regulating the expression of TEs, which have profound effects on genome stability and genetic imprinting (64).

Our study revealed several important hypermethylated DMGs that impact plant vacuole function, including proton ATPase type V, syntaxin-81, and 1-phosphatidylinositol-3-phosphate 5-kinase (65–67). Disruption in the transcription of these genes could potentially influence the plant’s susceptibility to pathogen infection. Notably, viroid infections often exert an effect on ATP-related processes (68). Among the hypermethylated DMGs, ATP binding emerged as the most significantly enriched term in the GO analysis (Fig. 5A). Conversely, GO analysis of hypomethylated DMGs with upregulation revealed enriched terms, such as ADP and ATP binding (Fig. 5B). However, the abundance of hypermethylated DMGs was 2.7-fold higher than that of hypomethylated DMGs.

Numerous protein-coding genes involved in metabolic pathways, such as arginine, proline, sphingolipids, ubiquinone, other terpenoids, and inositol phosphate, were hypermethylated in CBCVd-infected hop plants. Simultaneously, downregulation of hypermethylated DMGs associated with amino acid biosynthesis and glutathione metabolism was observed (Fig. 6A). Glutathione functions as a crucial antioxidant that safeguards cells against stress and disease-induced damage. Reduced synthesis of these molecules can heighten susceptibility to stress. Several studies have demonstrated that viroid infections in different hosts result in increased NADPH oxidase activity (19–21, 23, 25, 28, 69), leading to elevated levels of reactive oxygen species.

Interestingly, we found a homolog of oxylipin to be hypomethylated. The metabolism of these molecules is also believed to be linked to programmed cell death during plant-virus interactions (70, 71), in addition to their pivotal role in plant responses to stress, infection, and developmental processes (72). Oxylipin metabolism is also believed to be linked to programmed cell death during plant-virus interactions (70, 71). On the other hand, we have identified several hypomethylated protein-coding genes associated with arginine biosynthesis and nitrogen metabolism (Fig. 4B).

The GO analysis revealed that the maintenance of meristem identity was the most enriched biological process among the hypomethylated protein-coding genes (Fig. 4B). Hypomethylation of genes involved in primary plant cell wall biogenesis, cellulose synthesis processes, and growth regulation could significantly impact the host response, specifically in terms of growth maintenance and tissue regeneration. In CBCVd-infected hop plants, we discovered a hypomethylated homolog of cellulose synthase-like E1 protein, which is a crucial functional component for cell wall synthesis (73). Interestingly, altered expression of the cellulose synthase gene was also observed in tomato (*Solanum lycopersicum*) plants of the susceptible “Rutgers” cv infected with PSTVd (16, 69). However, among the downregulated and hypermethylated DMGs, we discovered a homologous gene encoding polygalacturonate-4-alpha-galacturonosyltransferase (PG4-alphaGal), which plays a key role in pectin biosynthesis (74).

In our study, we revealed two hypomethylated protein-coding genes that play important roles in DNA replication. The first gene, ribonuclease H2 subunit A, is involved in the degradation of RNA:DNA hybrids during DNA replication in humans (75). The second gene, factor MCM3, is crucial for the initiation of DNA replication and the formation of the pre-replication complex (76). Hypomethylation of such genes could potentially impact the stability of the hop genome when infected with CBCVd.

RNA polymerase activity is essential for transcribing genetic information from DNA to RNA. In our study, among the hypermethylated protein-coding genes, we discovered the rpac1 subunit of DNA-dependent RNA polymerases I and III. This subunit plays a crucial role in the synthesis of ribosomal precursor RNAs and small RNAs, including 5S rRNA and transfer RNAs (77). Interestingly, the GO analysis of hypermethylated DMGs revealed terms related to RNA polymerase complexes IV, II, and V. Notably, Pol II is also involved in viroid replication (78). This finding supports the idea that viroids induce activity in the RdDM pathway, thereby influencing host DNA methylation. It is possible that reduced RNA polymerase II activity could have implications for the host defense mechanism, leading to a differential replication and accumulation of CBCVd while affecting Pol IV and Pol V activities supporting the idea that viroids interfere with RdDM, thereby influencing host DNA methylation. These results also suggest that the host may inhibit the transcription of RdDM genes to protect itself against the viroid’s influence through the RdDM pathway. However, in our previous study, we did not observe any alterations in DRM gene expression in CBCVd-infected hop plants (37).

We observed hypomethylation of homologous protein-coding genes in CBCVd-infected hop plants. These genes include subunit 5 of the THO complex, the SEC31 transport protein homolog B, and the DnaJ protein homolog 2 and are known to play a role in RNA control (79), as well as protein transfer from the endoplasmic reticulum to the Golgi apparatus (80, 81). Interestingly, the DnaJ homolog 2 is also involved in the basal resistance to the rice blast fungus *Magnaporthe oryzae* (82). Additionally, we observed hypomethylation of a protein-coding gene for the E2 34 homolog of the ubiquitin-conjugating enzyme E2 34. This gene is required for the binding of ubiquitin (Ub) to cellular proteins in humans (83).

In the eukaryotic system, the transcriptional activity of Pol II relies on interaction with a mediator complex (MED) (84), which plays a crucial role in viroid replication. Viroid infection in hop plants has been demonstrated to lead to distinct regulatory responses in MED subunits, highlighting their involvement in plant defense mechanisms (85). Our findings revealed the hypomethylation of a homologous gene encoding MED7a. This observation suggests that CBCVd infection can activate the host factor genes necessary for viroid replication, providing further insights into the replication mechanism of viroids.

Previous studies have begun to demonstrate the impact of viroids on ribosomal processes in infected plants (16, 20, 21, 26, 86, 87). Notably, demethylation of certain rRNA genes has been observed in susceptible cucumber cv, such as “Suyo” (31) and “Marketer” (88), as well as in *N. benthamiana* (32). In these studies, the demethylation of rRNA genes correlated with their upregulation. However, these studies used HSVd, and the susceptibility of the cucumber cv “Marketer” to viroid infection has yet to be determined. In contrast, our study revealed the downregulation and hypermethylation of genes encoding 60S ribosomal protein L21-2, 40S ribosomal protein S10-1, and 60S ribosomal protein L35a-1, providing novel insights into ribosomal stress related to viroid infection.

Numerous studies have consistently demonstrated the impact of viroid infection on the regulation of R genes involved in plant defense mechanisms. These include resistance proteins such as the resistance protein to TMV (21) and the RPM1 (16). Additionally, the infection influences the expression of DRLs, resistance gene analogs (RGAs), and other proteins that interact with NBS-LRRs (22–29). Furthermore, it has been observed that LRR proteins are associated with jasmonates and ethylene-dependent systemic resistance, further emphasizing their role in plant defense responses (89).

Various studies have also reported the induction of protein kinases, specifically MAPK genes, and calcium signaling in relation to viroid pathogenesis (16, 19, 23, 28). In line with these findings, our study reveals that some of these protein-coding genes, such as MAPK5 and LRR receptor-like serine/threonine protein kinase, exhibit hypermethylation. However, the exact connection between their hypermethylation, resulting in inhibited transcription, and the plant defense mechanism remains unclear. Further investigation is required to elucidate the functional implications of these epigenetic modifications in the context of viroid-induced plant defense responses.

In contrast, our GO analysis based on 33 upregulated and hypomethylated DMGs in CBCVd-infected hop plants revealed a significant enrichment of processes related to plant-pathogen interaction activity, MAPK pathway activity, and plant hormone signal transduction activity (Fig. 6A). These findings are consistent with the previously mentioned transcriptome study (20). Specifically, we identified hypomethylated homologs of protein-coding genes associated with these processes, which exhibited upregulation in our data set.

For instance, we observed upregulation of the BAK1 protein and the FLS2 protein, which are known to form a complex involved in modulating abscisic acid (ABA) signal transduction (90), in citrus infected with CEVd (28). Consistently, we found a hypomethylated gene encoding RPM1, which is expected, given its association with viroid research (16). Furthermore, we identified a homolog for the LRK10L1.2 gene, which is known to positively regulate ABA signaling (91), among the upregulated and hypomethylated DMGs. Interestingly, our study also revealed that the hypomethylated RPM1 and RAR1 genes exhibited upregulation. These findings align with previous studies linking RPM1 to viroid infection responses (16) and the role of RAR1 in the resistance response of various plant species (92, 93). Additionally, we identified a protein-coding gene associated with N-mediated resistance against TMV, which plays a crucial role in the induction of resistance-related genes and the defense against TMV in tobacco plants through TIR signaling (94).

However, within the group of downregulated and hypermethylated DMGs, we discovered homologous genes involved in the positive regulation of defense responses against insects, specifically subtilisin-like proteases (SBT3). These proteases play a vital role in plant pathogen recognition and immune response (95), and their association with viroids has been reported in previous studies (96, 97). Furthermore, it is intriguing that among the downregulated and hypermethylated DMGs, we observed the presence of homologs of plant thaumatin-like proteins. These are categorized as PR5s and are involved in host defense and developmental processes in plants (98) but are also induced by viroids (99).

The analysis of enriched GO terms offers valuable insights into the investigation of DNA methylation patterns and the identification of associated genes and their expression patterns related to viroid pathogenesis. The relationship between differential DNA methylation and differential gene expression in CBCVd-infected hop plants is becoming more evident, as evidenced by the differential expression of protein-coding genes associated with processes characteristic of viroid infection. These findings emphasize the significant role of DNA methylation changes in the plant immune response to viroid infection. Further exploration of these mechanisms will contribute to a comprehensive understanding of the intricate interplay between DNA methylation, gene expression, and plant defense mechanisms in response to viroid infections.

## SUPPLEMENTAL MATERIAL

Supplemental tables spectrum.00394-24-s0001.xlsx Tables S1 to S6.

ASM does not own the copyrights to Supplemental Material that may be linked to, or accessed through, an article. The authors have granted ASM a non-exclusive, world-wide license to publish the Supplemental Material files. Please contact the corresponding author directly for reuse.
